# Prevalence of incidental thyroid malignancy on routine ^18^F-fluorodeoxyglucose PET-CT in a large teaching hospital

**DOI:** 10.1186/s41824-020-00089-5

**Published:** 2020-11-16

**Authors:** Shea Roddy, Thomas Biggans, Ahmad K. Raofi, Avinash Kanodia, Thiru Sudarshan, Prasad Guntur Ramkumar

**Affiliations:** 1 Department of Radiology, Ninewells Hospital, James Arrott Drive, Dundee, DD2 1SY Scotland, UK; 2 Department of Nuclear Medicine, Ninewells Hospital, James Arrott Drive, Dundee, DD2 1SY Scotland, UK

**Keywords:** Thyroid malignancy, Hybrid imaging, Positron emission tomography computed tomography, 18-Fluorodeoxyglucose, Standardised uptake value

## Abstract

**Purpose:**

To quantify incidental thyroid pathology including malignancy on routine ^18^F-FDG PET-CT scans

To compare standardised uptake values (SUV_max_) in thyroid malignancy subtypes

**Methods and materials:**

This is a retrospective study of all ^18^F-FDG PET-CT scans (*n* = 6179) performed in a teaching hospital between June 2010 and May 2019. RIS database search of reports for the word “thyroid” was performed. Studies with evidence of thyroid uptake were included. Patient age and gender, primary indication for PET scan (malignant or non-malignant), thyroid result on PET (diffuse or focal tracer uptake, SUV_max_), ultrasound and FNAC results were recorded.

**Results:**

Incidental abnormal thyroid tracer uptake as a proportion of all ^18^F-FDG PET-CT scans was 4.37% (*n* = 270). Out of region patients (*n* = 87) whose records could not be obtained were excluded leaving a study group of *n* = 183. Ninety-four in this group had focal uptake, and 89 had diffuse uptake. Fifty-five patients in the focal group had undergone further investigations. Of these, 30 were thought to be benign on USS alone, and 25 patients underwent USS/FNAC. Thirteen (24%) malignancies were identified (5 papillary, 6 follicular, 1 poorly differentiated thyroid cancer, 1 metastatic malignancy). Mean SUV_max_ for papillary carcinoma was noted to be 8.2 g/ml, and follicular carcinoma was 12.6 g/ml.

**Conclusion:**

Incidental abnormal thyroid ^18^F-FDG PET-CT uptake in PET-CT scans of 4.37% is in keeping with the known limited literature. Rather similar number of patients was noted in the focal and diffuse tracer uptake categories in the final study group. Around quarter of the focal lesions were identified to be malignant, implying focal lesions should always be further investigated.

## Introduction

The increasing role of PET-CT in oncological and non-oncological conditions has resulted in an increased detection of PET-CT incidentalomas, commonly involving the thyroid gland (The Royal College of radiologists, 2012; Delivanis & Castro, 2018; Vassiliadi & Tsagarakis, 2011).

**Fig. 1:**
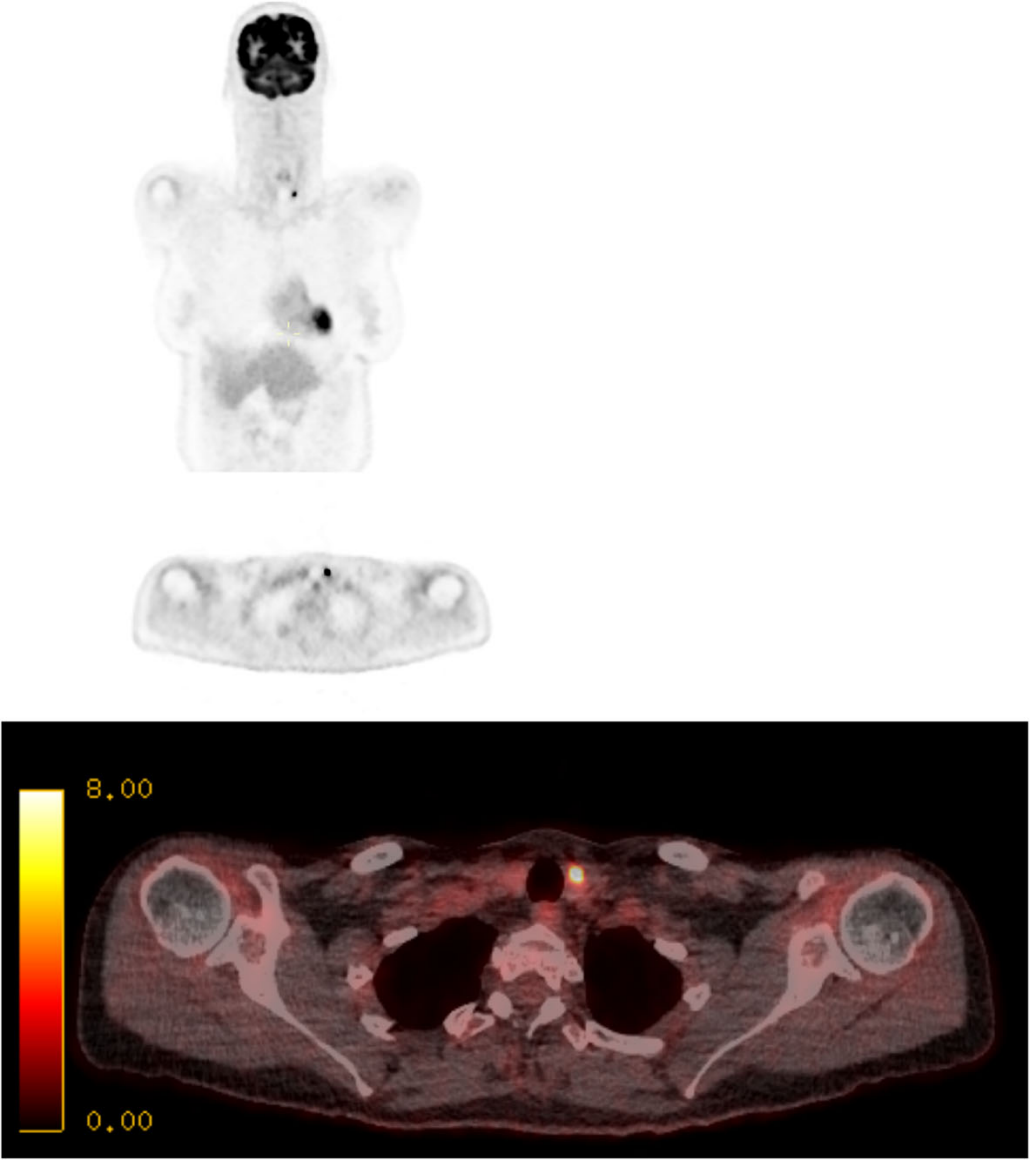
Fifty-three-year-old female with a diagnosis of Hodgkin’s lymphoma underwent PET-CT scan to monitor her response to chemotherapy. PET coronal (**a**) and axial (**b**) images show focus of increased tracer uptake in the left lobe of the thyroid. Image **c** shows fused axial PET-CT image showing the same abnormality (SUV max 11.1g/ml). FNAC biopsy showed this to be papillary thyroid carcinoma

**Fig. 2:**
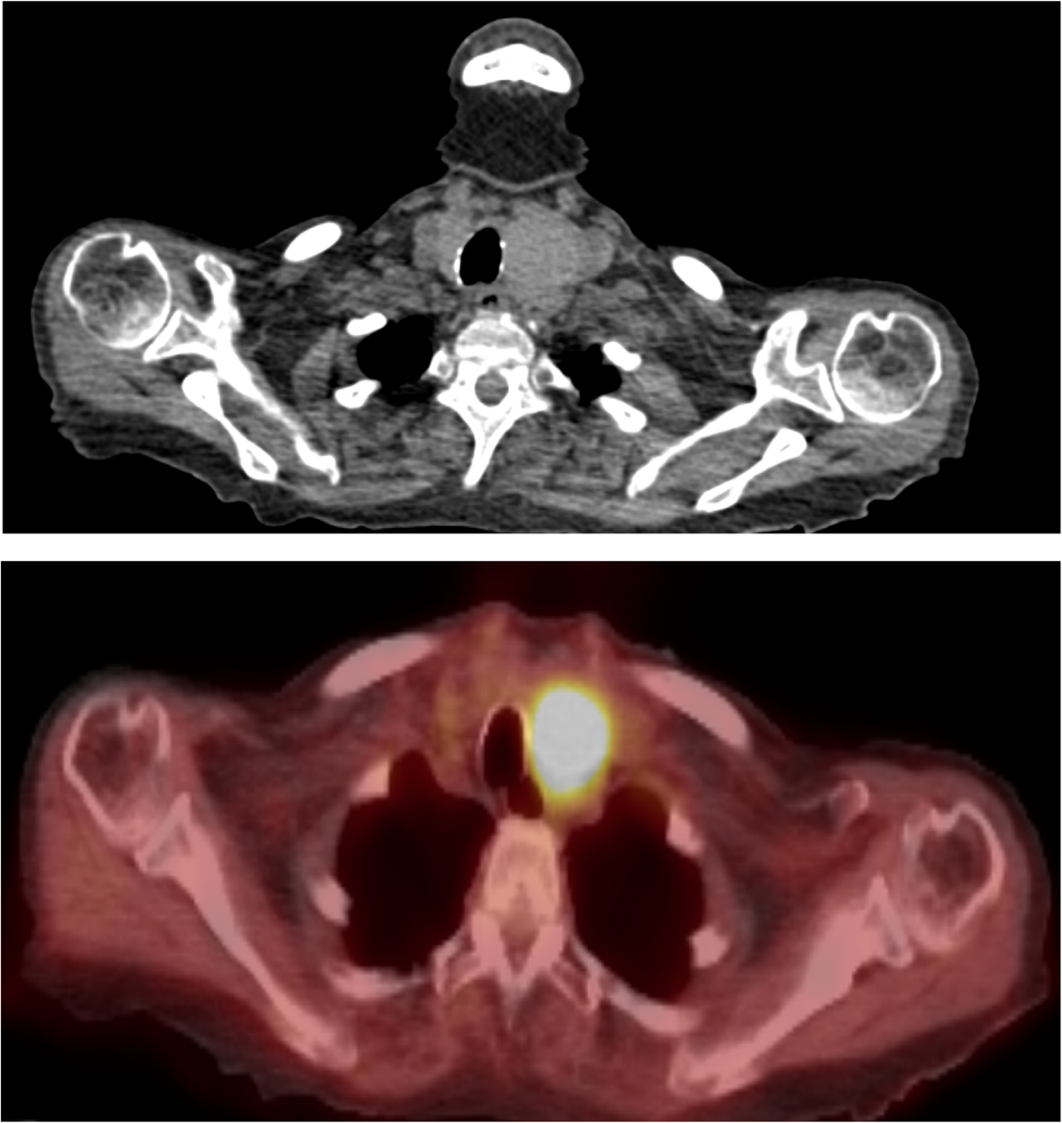
Seventy-nine-year-old female with a past medical history of systemic sclerosis and Raynaud’s disease. She presented with symptoms of weight loss, retrosternal chest discomfort and general cognitive decline. Prior investigations had shown a raised CRP and normal WCC in her routine blood, and an OGD showed oesophagitis only. She had a CT CAP and PET-CT to investigate for possibility of an organ tumour/large vessel vasculitis. Image **a** shows axial CT with a large left-sided thyroid mass. Image **b** is a fused axial PET-CT scan showing 33 mm × 36 mm metabolically active lesion (SUV max 10.3 g/ml) which occupies much of the left lobe of the thyroid gland and is responsible for tracheal deviation to the right. FNAC confirmed follicular carcinoma

The tracer ^18^F-FDG^1^ used in PET-CT can incidentally accumulate in the thyroid gland, either diffusely or focally. Incidental focal ^18^F-FDG uptake within the thyroid gland has previously been found to occur in 1.2–4.3% of all PET-CT scans in patients scanned for an alternative indication (Kao et al., 2012; Soelberg et al., 2012; Cohen et al., 2001; Kang et al., 2003; Chen et al., 2005; Chen et al., 2009; Ho et al., 2011). Patients with focal uptake within the thyroid gland are at a higher risk of malignancy, with studies reporting between 26 and 50% (Kao et al., 2012; Soelberg et al., 2012; Cohen et al., 2001; Chen et al., 2005; Chen et al., 2009; Kim et al., 2005; Chu et al., 2006; Bae et al., 2009). Figures 1 and 2 are two patients in our institution scanned for an alternative indication who subsequently were found to have thyroid malignancy. Further investigation of focal ^18^F-FDG uptake with ultrasound and FNAC^2^ is recommended because of this increased risk of malignancy (Pencharz et al., 2018; Haugen et al., 2015; Hoang et al., 2015).

PET-CT can deduce semi-quantitative evaluations of glucose metabolism in tissues by measuring standardised uptake value (SUV_max_) (Chu et al., 2014). The role of SUV_max_ in thyroid malignancy is debated as studies have produced conflicting results, and, although reasonable to suggest that malignancy is associated with a higher SUV_max_ value (Kumar et al., 2013), some studies have shown no correlation (Eloy et al., 2009; Are et al., 2007).

Although the usefulness of SUV_max_ in thyroid malignancy is questioned, a cytological diagnosis of focal thyroid FDG-PET incidentalomas is necessary considering the increased risk of malignancy.

The primary aim of our study was to quantify incidental thyroid malignancy on ^18^F-FDG PET-CT in patients scanned for an alternative indication. A secondary outcome was to compare mean, median, and range of SUV_max_ according to thyroid malignancy subtypes.

## Methods and materials

This is a retrospective study of all ^18^F-FDG PET-CT scans performed in a large teaching hospital between June 2010 and May 2019. We performed a CRIS (CDN Radiology Information System) database search, and results were filtered to include the word “thyroid” within the scan reports. All ^18^F-FDG PET-CT scans in our institution are double reported by four experienced consultants, two of whom are also experienced in routine thyroid imaging. Studies with no thyroid uptake were excluded. We manually collected patient’s data including age and gender, primary indication for PET-CT scan (malignant or non-malignant), thyroid result on PET (diffuse or focal uptake of ^18^F-FDG), ultrasound result and result of FNAC. Total number of FNAC diagnosed as thyroid malignancy and mean SUV_max_ values of thyroid malignancy subtypes were collected. Those patients who had tracer uptake but were lost to follow-up were excluded.

## Results

Six thousand, one-hundred and seventy-nine (*n* = 6179) ^18^F-FDG PET scans were performed during study period. Three-hundred and forty-two (*n* = 342) results contained the word “thyroid” of which two-hundred and seventy (*n* = 270) scans had increased uptake in thyroid gland. Eighty-seven (*n* = 87) patients were excluded (out of region patients, deaths, lost to follow-up). This left a study group of one-hundred and eighty-three (*n* = 183) of which one hundred and twenty-seven (*n* = 127) were females, and fifty-six (*n* = 56) were males. Ninety-four (*n* = 94) PET-CT scans had focal uptake, and eighty-nine (*n* = 89) had diffuse uptake. In our study group, those with diffuse uptake included seventy female patients (*n* = 70) and nineteen (*n* = 19) male patients. Fifty-seven females (*n* = 57) and thirty-seven male patients (*n* = 37) had focal uptake in our study group. Fifty patients in the focal group underwent further investigations. Of these, 30 were thought to be benign on USS alone and 25 patients underwent USS/FNAC, where 52% (52%, *n* = 13/25) were confirmed to be malignancy. Thyroid malignancy subtypes included papillary (*n* = 5), follicular (*n* = 6), metastatic malignancy (*n* = 1) and poorly differentiated carcinoma of the thyroid (*n* = 1). Patients who had a previous history of thyroid cancer with increased uptake were included in the study (*n* = 1). Eight (8/25) pathology result confirmed benign lesions including non-neoplastic (*n* = 2), follicular with Hurthle cell type changes (*n* = 1), and colloid nodule (*n* = 5). The remainder of the FNA biopsies were deemed indeterminate (*n* = 4/25) by the reporting pathologist giving a malignancy positive rate of 62% (13/21) within the confirmed results. Overall, 13 malignancies were identified out of the 55 patients with focal uptake who underwent further assessment (24%).

Abnormal tracer uptake in the thyroid gland incidentally as a proportion of all ^18^F-FDG PET-CT scans was 4.37%. Abnormal focal tracer uptake as a proportion of all ^18^F-FDG PET-CT scans was 2.77%. Mean SUV_max_ in focal malignant lesions ranged from 4 to 35.36 g/ml (Table 1) and in focal benign lesions ranged from 1.6 to 18.2 g/ml (Table 2). Mean SUV_max_ value for papillary carcinoma was 8.2 g/ml, and follicular carcinoma was 12.6 g/ml. Median SUV_max_ for papillary carcinoma was 7.4 g/ml, and follicular carcinoma was 8.2 g/ml. Given the small sample size and significant overlap in the obtained SUV_max_ data for the two groups, a threshold with a clinically valuable power cannot be obtained to differentiate benign and malignant tracer uptake and hence is not stated here.

**Table 1:** The SUVmax values for the FNAC-proven malignancies

Type of malignancy	SUV value (g/ml)
**Papillary**	11.1
**Papillary**	4
**Papillary**	5.4
**Papillary**	13.3
**Papillary**	7.4
**Follicular**	10.3
**Follicular**	7.7
**Follicular**	35.36
**Follicular**	6.7
**Follicular**	6.8
**Follicular**	8.7
**Metastatic malignancy**	4.8
**Poorly differentiated carcinoma of thyroid**	8.9

**Table 2:** The SUVmax values for the eight FNAC reported benign by the pathologist

Pathological report	SUVmax value (g/ml)
**Non-neoplastic**	18.2
**Non-neoplastic**	12.48
**Follicular adenoma with Hurthle cell type**	5.73
**Colloid**	7.45
**Colloid**	6.1
**Colloid**	1.6
**Colloid**	3.8
**Colloid**	5.8

## Discussion

Incidental abnormal thyroid ^18^F-FDG uptake as a proportion of all PET-CT scans and incidence of malignancy in focal abnormal thyroid ^18^F-FDG uptake in our patient group are both comparable with the available limited literature (Vassiliadi & Tsagarakis, 2011; Kao et al., 2012; Soelberg et al., 2012; Cohen et al., 2001; Kang et al., 2003; Chen et al., 2005; Chen et al., 2009; Yi et al., 2005; Ho et al., 2011; Kim et al., 2005; Chu et al., 2006), reiterating the importance of detecting and investigating focal thyroid uptake on ^18^F-FDG PET-CT. Around a quarter of focal thyroid uptake were malignant; hence, focal lesions should be investigated further, if clinically appropriate. A significant proportion of ^18^F-FDG PET-CT scans are performed for known/unknown malignancy, and further assessment of incidental thyroid lesions may not be possible for many reasons. Regardless, it is important to detect thyroid lesions in all patients in order to optimise appropriate clinical decision-making.

Some of the patients who underwent USS were deemed benign sonographically despite having focal thyroid tracer uptake. It is recommended that all thyroid tracer focal uptake, if clinically appropriate, are investigated further with an ultrasound and FNAC because of the increased risk of malignancy (Bae et al., 2009; Pencharz et al., 2018; Haugen et al., 2015). Currently, we do not have agreed local guidelines for investigation of focal thyroid uptake on ^18^F-FDG PET-CT, but based on current recommendations and findings in this study, it would be our future practice to investigate all focal lesions to undergo FNAC where clinically necessary.

Similar number of diffuse and focal thyroid tracer uptake were found in our study, and this is important to differentiate them as they have different outcomes for patients. Abnormal thyroid tracer uptake, diffuse or focal, also appears to be more in females, and this is comparable with current literature (Stangierski et al., 2014).

Our secondary aim was to compare SUV_max_ across thyroid malignancy histological subtypes. Follicular carcinoma has a higher mean, median and range of SUV_max_ than papillary carcinoma in this study. Soelberg et al. in their meta-analysis found mean SUV_max_ to be 6.9 g/ml in malignant lesions (Soelberg et al., 2012), lower than our findings for both follicular and papillary carcinoma but comparable with our median SUV_max_. Our sample size for each malignancy subtype is low, and mean SUV_max_ and median SUV_max_ might be affected by this, so should be interpreted cautiously.

Our study found malignancies with a wide range of SUV_max_ values, implying a higher mean SUV_max_ does not necessitate malignancy. It is debatable if SUV_max_ can differentiate between benign and malignant lesions, but overall it is thought that mean SUV_max_ is lower in benign lesions compared to malignant lesions (Soelberg et al., 2012). It is also unclear if a specific SUV_max_ indicates malignancy and certain cutoffs have been stipulated. In our study, we were unable to confidently identify a cutoff point for SUV_max_ to identify malignancy due to considerable overlap in SUV_max_ values in both benign and malignant focal uptake lesions. This is echoed by the results of two smaller studies, which also showed a large overlap in SUV_max_ value between benign and malignant lesions (Brindle et al., 2014; Agrawal et al., 2015). With the findings in this study and other studies, it is difficult to confidently use mean SUV_max_ value alone in differentiating between benign and malignant thyroid lesions. There is a role for SUV_max_ in thyroid malignancy, but a specific defined value or range cannot be accurately established in differentiating between benign or malignant lesions.

There are a few limitations to our study that we acknowledge. Our biggest limitation was that we excluded nearly a third of patients with focal thyroid tracer uptake who were lost to our follow-up. Most of those patients were referred to us initially from regional centres, and their follow-up records or results were not available for our reference. Despite this, the study still showed the prevalence of incidental thyroid uptake on ^18^F-FDG PET-CT. Another limitation of our study is that only a word search for “thyroid” was done in our reports, and images were not reviewed. As described earlier, all our scans are double reported within a pool of 4 experienced consultants, two of whom specialise in thyroid imaging. Most of these patients undergo scans for known malignancy, and a significant proportion of these scans are reviewed again for MDT requirements. Furthermore, there is a dedicated discrepancy meeting to identify reporting errors. We strongly believe, given the above, that the possibility of missing unreported thyroid lesions in these scans to be very low.

## Conclusion

Standardised approach needed for investigation of incidental ^18^F-FDG PET-CT focal uptake in the thyroid gland due to high prevalence of malignancy and a combination of ultrasound with FNAC is advised.

There is conflicting evidence at how to utilise SUV_max_ in focal thyroid tracer uptake, but its use in combination with ultrasound and histopathological findings should be sought with further bigger studies. We established that SUV_max_ is relatively higher in follicular carcinoma than papillary carcinoma; however, further research in large patient groups is needed.

## Data Availability

The datasets used and/or analysed during the current study are available from the corresponding author on reasonable request

## Footnotes

1 ^18^F-fluorodeoxyglucose

2 Fine needle aspiration with cytology

## References

[CR1] Agrawal K, Weaver J, Ul-Hassan F, Jeannon JP, Simo R, Carroll P, et al. Incidence and significance of incidental focal thyroid uptake on (18)F-FDG PET study in a large patient cohort: retrospective single-Centre experience in the United Kingdom. Eur Thyroid J 2015; 4:115-122. doi: https://doi.org/10.1159/000431319 10.1159/000431319 PMC4521059 26279997

[CR2] Are C, Hsu JF, Schoder H, Shah JP, Larson SM (2007). FDG-PET detected thyroid incidentalomas: need for further investigation?. Ann Surg Oncol.

[CR3] Bae JS, Chae BJ, Park WC, Kim JS, Kim SH (2009). Incidental thyroid lesions detected by FDG-PET/CT: prevalence and risk of thyroid cancer. World J Surg Oncol.

[CR4] Brindle R, Mullan D, Yap BK, Gandhi A (2014). Thyroid incidentalomas discovered on positron emission tomography CT scanning - malignancy rate and significance of standardised uptake values. Eur J Surg Oncol.

[CR5] Chen W, Parsons M, Torigian DA, Zhuang H, Alavi A (2009). Evaluation of thyroid FDG uptake incidentally identified on FDG-PET/CT imaging. Nuclear Med Commun.

[CR6] Chen YK, Ding HJ, Chen KT, Chen YL, Liao AC (2005). Prevalence and risk of cancer of focal thyroid incidentaloma identified by 18F-fluorodeoxyglucose positron emission tomography for cancer screening in healthy subjects. Anticancer Res.

[CR7] Chu QD, Connor MS, Lilien DL, Johnson LW, Turnage RH (2006). Positron emission tomography (PET) positive thyroid incidentaloma: the risk of malignancy observed in a tertiary referral center. Am Surg.

[CR8] Chu Y, Zheng A, Wang F, Lin W, Yang X, Han L (2014). Diagnostic value of 18F-FDG-PET or PET-CT in recurrent cervical cancer: a systematic review and meta-analysis. Nuclear Med Commun.

[CR9] Cohen MS, Arslan N, Dehdashti F, Doherty GM, Lairmore TC (2001). Risk of malignancy in thyroid incidentalomas identified by fluorodeoxyglucose-positron emission tomography. Surgery.

[CR10] Delivanis DA, Castro MR (2018) Thyroid nodules. Humana press; Cham, Switzerland: 2017. Thyroid Incidentalomas; pp. 153–167.

[CR11] Eloy JA, Brett EM, Fatterpekar GM, Kostakoglu L, Som PM, Desai SC, Genden EM (2009). The significance and management of incidental [^18^F]fluorodeoxyglucose–positron-emission tomography uptake in the thyroid gland in patients with cancer. Am J Neuroradiol.

[CR12] Haugen BR, Alexander EK, Bible KC, Doherty GM, Mandel SJ, Nikiforov YE (2016). 2015 American Thyroid Association management guidelines for adult patients with thyroid nodules and differentiated thyroid cancer: the American Thyroid Association guidelines task force on thyroid nodules and differentiated thyroid cancer. Thyroid.

[CR13] Ho TY, Liou MJ, Lin KJ, Yen TC (2011). Prevalence and significance of thyroid uptake detected by ^18^F-FDG PET. Endocrine..

[CR14] Hoang JK, Langer JE, Middleton WD, Wu CC, Hammers LW, Cronan JJ, et al. Managing incidental thyroid nodules detected on imaging: white paper of the ACR incidental thyroid findings committee. J Am Coll Radiol 2015; 12: 143–150. doi: https://doi. org/10.1016/j.jacr.2014.09.038 10.1016/j.jacr.2014.09.038 25456025

[CR15] Kang KW, Kim SK, Kang HS, Lee ES, Sim JS (2003). Prevalence and risk of cancer of focal thyroid incidentaloma identified by 18F-fluorodeoxyglucose positron emission tomography for metastasis evaluation and cancer screening in healthy subjects. J Clin Endocrinol Metabol.

[CR16] Kao YH, Lim SS, Ong SC, Padhy AK (2012). Thyroid incidentalomas on fluorine-18-fluorodeoxyglucose positron emission tomography-computed tomography: incidence, malignancy risk, and comparison of standardized uptake values. Cancer Assoc Radiol J.

[CR17] Kim TY, Kim WB, Ryu JS, Gong G, Hong SJ (2005). 18F-fluorodeoxyglucose uptake in thyroid from positron emission tomogram (PET) for evaluation in cancer patients: high prevalence of malignancy in thyroid PET incidentaloma. Laryngoscope.

[CR18] Kumar V, Nath K, Berman CG, Kim J, Tanvetyanon T (2013). Variance of SUVs for FDG-PET/CT is greater in clinical practice than under ideal study settings. Clin Nucl Med.

[CR19] Pencharz D, Nathan M, Wagner TL (2018). Evidence-based management of incidental focal uptake of fluorodeoxyglucose on PET-CT. Br J Radiol.

[CR20] Soelberg KK, Bonnema SJ, Brix TH, Hegedüs L (2012). Risk of malignancy in thyroid incidentalomas detected by 18F-fluorodeoxyglucose positron emission tomography: a systematic review. Thyroid.

[CR21] Stangierski A, Woliński K, Czepczyński R, Czarnywojtek A, Lodyga M, Wyszomirska A, et al. The usefulness of standardized uptake value in differentiation between benign and malignant thyroid lesions detected incidentally in 18F-FDG PET/CT examination. PLoS One 2014; 9: e109612. doi: https://doi.org/10.1371/journal.pone.0109612 10.1371/journal.pone.0109612 PMC4190406 25296297

[CR22] The Royal College of radiologists (2012). iRefer: making the best use of clinical radiology.

[CR23] Vassiliadi D.A., Tsagarakis S. Endocrine incidentalomas—challenges imposed by incidentally discovered lesions. National review of endocrinology, 2011; June 28^th^; 7:668–680. Doi: 10.1038/nrendo.2011.92 10.1038/nrendo.2011.92 21709710

[CR24] Yi JG, Marom EM, Munden RF, Truong MT, Macapinlac HA (2005). Focal uptake of fluorodeoxyglucose by the thyroid in patients undergoing initial disease staging with combined PET/CT for non–small cell lung cancer. Radiology.

